# The Impact of COVID on Adolescent Mental Health, Self-Harm and Suicide: How Can Primary Care Provider Respond? A Position Paper of the European Academy of Pediatrics

**DOI:** 10.3389/fped.2022.800000

**Published:** 2022-03-23

**Authors:** Pierre-André Michaud, Laurent Michaud, Artur Mazur, Adamos Hadjipanayis, Carole Kapp, Anne-Emmanuelle Ambresin

**Affiliations:** ^1^Lausanne University Hospital, Lausanne, Switzerland; ^2^Department of Psychiatry, Lausanne University Hospital, Lausanne, Switzerland; ^3^Faculty of Medicine, University of Rzeszów, Rzeszów, Poland; ^4^Medical School, European University of Cyprus, Nicosia, Cyprus; ^5^Department of Pediatrics, Lausanne University Hospital, Lausanne, Switzerland

**Keywords:** adolescent, suicide, self-harm, mental health care, COVID

## Abstract

Adolescents aged 10 to 19 live a period of their life marked by opportunities and vulnerabilities during which the issue of mental health is of prime importance. Since several decades, and especially since the start of the COVID pandemic, mental health problems and disorders among adolescents have increased around the world. Depression, self-harm and suicidal behavior are common during this period of life, and pediatricians can play a pivotal role in identifying affected or vulnerable youngsters. This article describes risk factors for self-harm and suicidal behavior and reviews how primary care pediatricians and health professionals can respond to such situations. This scoping review is based on existing evidences as well as the authors clinical experience. It suggests concrete actions that can be taken to secure the life of at risk teenagers, and discusses how to organize the transfer to mental health professionals when needed.

## Introduction

The period of adolescence represents a crucial phase of life during which the individual undergo a rapid set of physical and psychosocial changes and as such constitutes a period of opportunities and threats to successful development (1, 2). The importance of mental health is in this respect undebatable, as wellbeing and a good mental health balance improves the quality of life on the long term and decreases the odds for poor family, educational and social outcomes (2). For several years, many institutions and authors have called attention to the personal and social menace that the growing proportion of young people suffering from mental health burden or disorders signifies for the future of our societies (3–5). The huge transformation that the brain undergoes over this period (6, 7) explains why many mental health problems or disorders tend to appear during adolescence, such as schizophrenia or bipolar disorders, or more commonly anxiety and depression. As a result, it comes as no surprise that self-harm and suicidal behavior appear during adolescence. They thus represent a major challenge for health care providers. Self-harm can be defined as intentional injury of one's body (often the skin) not necessarily linked with suicidal intention; it includes gestures such as poisoning, cutting oneself with a knife or a sharp object, or other kinds of self-mutilations (8, 9). The most common form of self-harm is using a sharp object to cut the skin. Even if non-suicidal self-injuries (NSSI) and suicide attempts are considered as separate entities in the DSM V (8, 9), it is not always easy to distinguish between them, as the intention of the adolescent (conscious or unconscious) is not easily perceptible; this leads some authors to consider all these behavior along a continuum from NSSI as a relief of tension without any intention to kill oneself to suicide attempt (8).

According to a recent publication (10), adolescent suicide rates have, on average, declined slightly over the past two decades in Europe during 2000's [this is not the case in USA (11)]. However, suicides constitute in many countries the second leading cause of death after road injuries (12). Beyond death by suicide, there are many more young people who engage in suicidal behavior, and the estimate is that for every death by suicide there are ten more attempts, part of them being undisclosed (13). In addition, the impact of suicidal behavior on relatives, especially parents, brothers and sisters, cannot be ignored (14).

## The COVID pandemics: A Severe Impact on Adolescent Mental Health

As soon as during the spring 2020, several publications have suggested that the COVID pandemics had an important negative impact on the life and the mental health of children and adolescents (15–17). This has been confirmed over the next months until the middle of 2021 (18–23). Symptoms such as increased depression and anxiety are the more common reported issues, but an extremely worrying observation is a rise in suicide attempts, which is confirmed in anecdotal observations as well as preliminary investigations (19, 20, 24–29). Despite the publication of a recent non peer-reviewed study which suggests no raise in death by suicide among UK adolescents (30), it is presently still not possible to assess the impact of COVID on deaths by suicide among adolescents in Europe.

There are several reasons for an impact of COVID on mental health: the most important one is probably the one of lockdown and school closure. Especially within families of lower socio economic status, having to live and work without any or with poor connection with peers, being unable to attend distance learning sessions because of the lack of internet tools impose a heavy load on the mood of children and young people (23). A limited access to sports activities and of opportunities for social gathering and exchanges with peers has added to the psychosocial load of the pandemics. Moreover, situations of family conflict and violence have increased (31), as can be easily understood under times of high pressure from governmental agencies. Another additional factor is the one of uncertainty about the potential consequences of a COVID infection on relatives (or oneself). In addition, the pandemic raises the issue of the broad contributing factors on the spread of the COVID such as the decline in biodiversity and the change and warming of the climate (32) all phenomenon's which impact on the mood and wellbeing of the younger generation and participates in the rising mental health burden (33, 34).

Lastly, access to mental health care has been reduced, with the closure or restrictive access to ambulatory care, as well as, in the early phase of the pandemic, the reluctance to consult linked with the fear to be infected (15, 21, 35). It is alas anticipated that the addition of all these factors will lead to long-lasting negative outcomes. This is to say that primary care providers, particularly pediatricians, even if this seems difficult for many of them (36) need to play a role in detecting mental health problems among their young patients and make efforts to mitigate these consequences, including the stimulation of protective factors such as supportive family, high self-esteem, good social network, etc. (9, 22, 35, 37–40). The purpose of this scoping review is to outline how they can identify depression, self-harm and suicidal behavior in their everyday practice, taking into account the availability of specialized services and institutions (2, 41, 42). The content is based both on available evidences and the clinical experience of the authors.

## How to Identify Depression, Self-Harm and Suicidal Behavior

It is part of the primary care of adolescents to perform, as far as time allows for, a thorough evaluation of the patient's lifestyle, and to assess both health enhancing and health damaging situations and behavior (43). Adolescents should thus be informed that any consultation is an opportunity to discuss issues about their health and lifestyles. The “HEEADSSS” acronym (44) is widely used in many countries to remember which important aspects of the adolescent's daily life can be assessed, as some of them constitute potential protective as risk factors for mental health burden and disorders. They include areas such as Home, Education and school, Eating habits, Activities (peers, sports, hobbies), Drugs, Sexuality, Safety practices and mental health, including potential Suicidal thoughts and behavior (45). In doing so, health professionals should not only focus on risk factors or risky behavior, but also on protective factors and resilience: quality of the relationship with parents, caregivers, teachers, mentors. Close friends who can provide support, structured peer activities such as sports club, investment in an attractive hobby, etc. This exploration is all the more useful because it provides clues as how the adolescent can overcome stressful situations, conflicts or bouts of depression.

This assessment can be performed during a health check-up, as part of the first encounter with the adolescent or at any other time (46). The practitioner should adapt the questions and language to the adolescent's developmental stage and proactively mention that some issue can remain confidential, if asked for by the adolescent (46–48). Hence, all adolescents should be interviewed alone, at least for a few minutes, to guarantee privacy and confidentiality, but the parents should be involved as much as possible, and invited to give their own view of the situation. Such a review does not necessarily take much time and can be spread over several occasions, for instance if the practitioner is in a hurry. As mentioned in the introduction, the COVID pandemics has increased the prevalence of mental health problems among adolescents, and it is important for the health care provider to systematically explore in the review of the patient's mental health several crucial issues as mentioned in Table 1: many adolescents are impacted by the fluctuating sanitary measures taken by health and political administrations, and these restrictions raise lots of anxieties and concerns that must be addressed.

**Table 1:** Issues to be discussed with the adolescent patient in the context of the COVID pandemics.

1	Explore the adolescent's knowledge and representations about the COVID
2	Provide evidence-based information on the transmission of COVID and its impact on health
3	Discuss protective behavior vis à vis COVID: hand-washing, wearing masks, distancing, etc.
4	Assess the impact of COVID on the adolescent's mental health: worries about the health of relatives, fears of being infected, depression as a result of isolation, fear around one's school and professional future, anxieties about the future of the world, etc.
5	Inquire about resilience factors and reasons for hope

## How to Assess Depression, Self-Harm and Suicidal Behavior

The assessment of mental health, e.g., mood, expectations, fantasies and behavior is an important part of the HEEADSSS review. It is all the more important because it is difficult for many adolescents to recognize their needs and to spontaneously disclose their suffering, their mental health burdens and particularly their suicidal thoughts or plans. Mental health disorders among adolescents, especially depression, are often covert and practitioners should be alerted by physical symptoms such as repeated pain, tiredness or sleep problems as well as unexpected violent behavior or drop in school grades. Many adolescent patients display so-called co-morbid symptoms (49), e.g., a mix of symptoms suggesting depression and physical complaints, or addictive behavior (substance misuse, internet overuse) and anxiety. While there are many screening questionnaires available, such as the Beck Depression Inventory (50), the busy pediatrician could ask simple questions, such as “do you have difficulties getting up in the morning?”, “do you often feel that you lack energy or courage?”, “do you have sleeping difficulties?”, “How is your appetite?”, “Have you lost interest in your daily activities?”, “Are you anxious about your future?”, Do you hurt yourself?”, etc. There are two important criteria that should raise the suspicion of a serious mental health problem: the first one is the duration of symptoms beyond a few weeks; the second is a major impact on daily life and activities, including isolation, lack of connectedness with peers, all symptoms that can be recognized by the adolescent's acquaintances. These two factors should prompt a thorough evaluation by a specialist.

Clinicians should *always* ask about suicidal thoughts and behavior when young patients exhibit symptoms of mental health problems (51). As already mentioned, the delimitation between NSSI and suicide attempts is not easy (9), as it is sometimes difficult for both the patient and the physician to gauge the intentionality of such a gesture; in addition NSSI, especially if repeated must be considered as a risk factor for suicide attempt or suicide. There are risk factors that should systematically be looked for when one suspects mental health problems (52), and which are listed in Table 2, among which past suicide attempt should be openly addressed. However, although they contribute to increasing the hazard for suicidal behavior, they should not be considered as an addition of risk or as an index, but rather should be appraised in the context of the patient and his environment. Unfortunately, it is still wrongly believed by many health professionals that tackling the issue of suicide may foster suicidal gesture. The opposite is true, e.g., discussing overtly suicide with young people does not increase the odds for suicide but may on the contrary decrease it.

**Table 2:** Risk and protective factors for suicidal conducts.

1.	Individual
	Self-harm, past suicide attempt (revealed or unrevealed)
	Depression, anxiety, misuse of alcohol & drugs, mental health disorder
	Traits: impulsivity, auto and hetero-aggression, violence
	Low self-esteem
	Somatic complaints
	LGBTIQ+ orientation
	Runaway.
2.	Family
	Past suicide among close relatives
	Lasting mental health disorders among close relatives
	Abuse and neglect (including sexual abuse)
	Family conflicts, violence, divorce
	Poor communication patterns
3.	Interpersonal and environmental
	Inter-personal losses, relationship break-ups, death of friends
	Peer rejection
	School problems and academic stress
	Bullying and cyber-bullying
	Contagion, imitation
4.	Protective factors (should not deter physician to take action)
	High self-esteem
	Internal locus of control
	Fluid communication with parents, support from parents and family
	Good social network

One factor that should not be forgotten is the one of imitation or “contagion” (52). Since several decades, we know that some events can increase the risk of suicide among adolescents: media reports, especially those glorifying the suicide of well-known artists have been well-recognized, especially if there are similarities between the adolescent and his “model.” Likewise, the death of a youngster within a school or a group can be “contagious” (53). More recently, it has been suspected that internet sites, especially those suggesting concrete methods to dye by suicide, may have a contributing effect on adolescent suicide proneness. To prevent this kind of contagion, so-called postvention interventions can be offered, such individual and group counseling to affected peers or screening high risk individuals through personal interviews. In addition, postvention procedures can also mitigate the traumatic impact of such event on the family and acquaintances.

Not all adolescents reporting suicidal ideation are at high risk for suicide or suicide attempt. In a recent multi-country pooled estimation of suicidal ideation among 13–17 year old adolescents, the percentages of those reporting thoughts varied from around 1 to 35%, with a mean around 11–12% among those aged 13–15 and around 18% among those aged 16–17 years, with a slight but significant difference between boys and girls (males >females). It is thus important for primary care providers to assess the risk for taking action and thus to decide whether to refer or not the adolescent to a mental health specialist or institution (9, 42, 54).

## Dealing with a Suicidal Crisis

**Figure 1:**
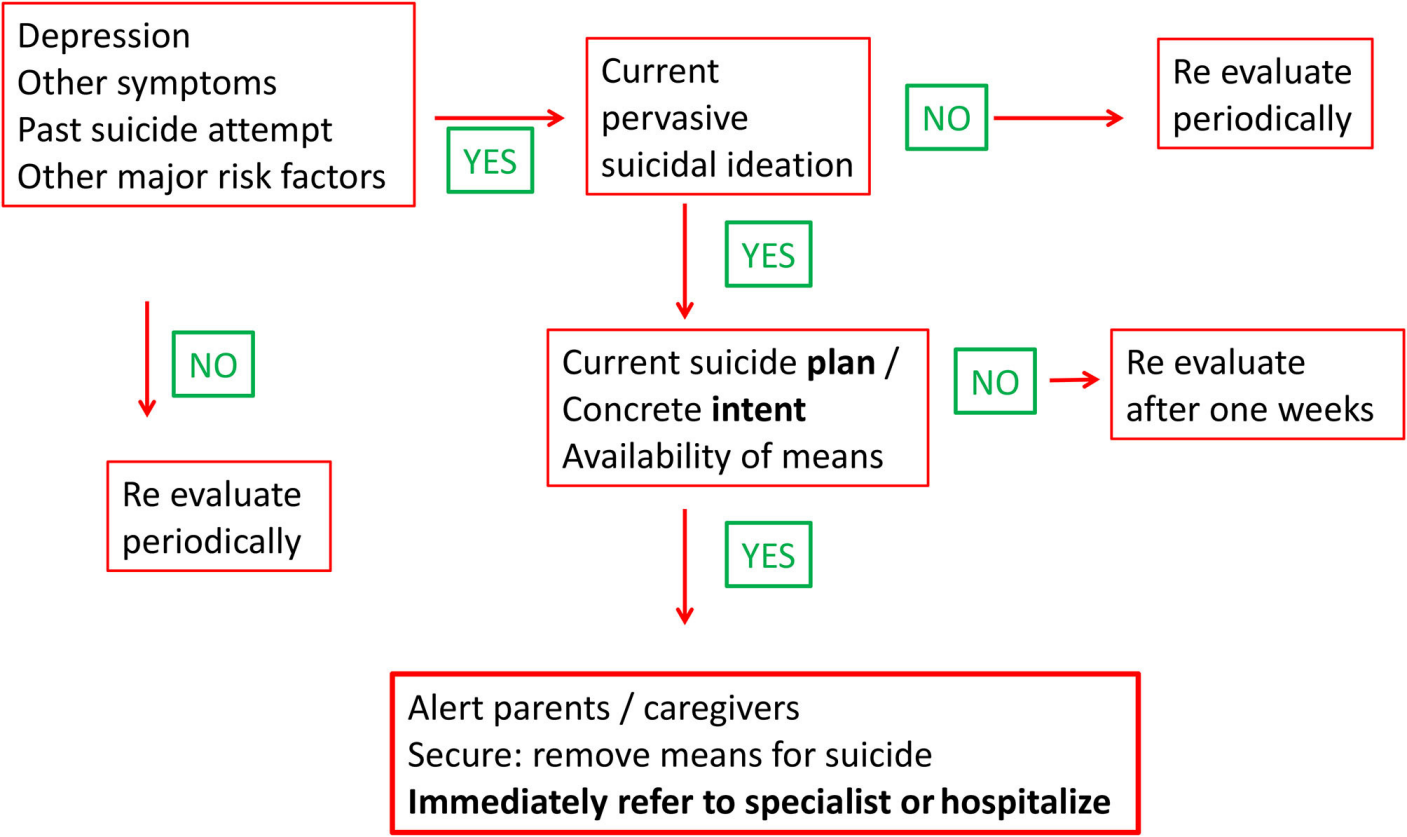
Step by step assessment of suicidal behavior and how to respond.

Figure 1 provides a tentative algorithm that should assist pediatricians in assessing suicidal adolescents and help them to decide whether to immediately refer to a psychiatrist or hospitalize the young patient. It does not represent an authentic risk assessment tool (55) but rather suggests how primary care providers should react when faced with situations pertaining to self-harm/suicide. When an adolescent mentions suicidal thoughts but does not disclose suicidal plans, it is usually safe to monitor the situation within a few days and explore carefully the family and the social context of the patient, e.g., the presence of trusted adults or peers with whom a stable connectedness has been established; In addition, the practitioner should discuss concretely with the adolescent what she/he can imagine or consider to improve the situation and negotiate an individualized follow-up plan. Situations with high risks of suicidal gesture are the ones of adolescents who tend to abuse alcohol or other psychotropic substances, who have recently tried to kill themselves and are still considering to do so, and, in addition, who mention immediate identified and available means or methods. Table 3 displays some of the issues that must be addressed by the health care provider. The patient, usually interviewed with the guarantee of confidentiality, should without fail be notified that in instances with immediate threat to life, this is no longer possible and that the parents or caregivers should be notified. This is all the more important as the parents, not rarely, are unaware of their children's thoughts about suicide and death. Some parents are unable to provide functional management to their adolescent, so that it is necessary under these circumstances to identify adults (e.g., relatives) who can take over the control for a while, or to refer to family therapists or social workers.

**Table 3:** Addressing situation with high risk of suicide.

1	Not jump on solutions and responses but first listen to the adolescent.
2	Thank the adolescent for his confidence and for sharing his situation.
3	Show empathy: “I am touched by your sadness, anger, etc.”
4	Discuss events that may have prompted the adolescent to consider suicide.
5	Review the adolescent's psychosocial resources (close friend, project for future.)
6	Explore and consider alternatives to suicidal behavior.
7	Explain why you need to break confidentiality and alert the parents and caregivers.
8	Remove in the adolescent's environment immediate means to dye from suicide (firearms, medication, rope.)
9	Develop a plan to secure the adolescent's life (e.g., not leaving the adolescent alone.)

In critical high risk situation, the most important measure to take is to ensure the short-term safety of the adolescent e.g., not leave her/him alone and removing potential means to die from suicide; this exploration should be carefully carried out and tackle all potential available means. Not all countries have a health care system that provides immediate access to specialized mental health facilities (41), and in some instance, this is not mandatory. If no psychiatrist is immediately available, with the support of the parents, sometimes the school staff, other trusted adults, it is possible to build a network and develop a plan that is accepted by the patient and other involved persons. The plan should include, with the active collaboration of the adolescent, the identification of the stakeholders, the phone number of the hospital's emergency, access to dedicated helplines, etc. In severe situations, and when it seems not possible to build such a response, the primary care provider's responsibility is to hospitalize the adolescent, either in a non-specialized or specialized ward, until a link with a mental health specialist can be established. Again, one important way to decrease the risk for suicide is to remove the access to the expected mean, with the support of surrounding responsible adults and to make sure that the patient is not left alone. It should be stressed that the simple fact that the adolescent has been able to share his situation and intent can decrease the odds for attempting or dying from suicide (56).

It is not rarely difficult to convince an adolescent disclosing high risk of self-harm to consult a mental health professional. In several European countries, being under the care of a psychologist or a psychiatrist raises stigmatizing reactions from the peers or surrounding adults and parents. The role of the primary care provider is to kindly accompany the young patient in the process, either by organizing a co-consultation with his/her colleague, or by explaining that undertaking such a process will not necessarily lead to a years-long treatment. It is also very important to involve the parents, especially when the situation is associated with a dysfunction of the family.

## Facing a Suicide Attempt

Not rarely, pediatricians are requested to intervene when adults – parents, teachers, social workers and friends – realize that a young person has attempted suicide. In some situation, such as hanging, wounds or drowning, the attempt needs emergency intervention. In many instances however, the young persons, especially the females have ingested medication, often of unknown amount, and not infrequently, the patient is still alert, especially when the surrounding persons learn about the attempt after a few hours or the day after. It is usually wise under these circumstances to still consider a hospitalization. Even if it is realized in a non-specialized unit, such a hospitalization stresses the seriousness of such a gesture and avoids the denial that often emerges, both from the adolescent himself and from the parents. It also allows to create a link with a psychotherapist and plan for an ambulatory follow-up. Some mental health facilities have developed effective structured program to deal with these situations, including symptoms of severe mental disorders such as delusions or endogenous depression (57).

## Conclusion: A Role to Play for Pediatricians

In many countries the training of pediatricians in the area of mental health and adolescent health is far from optimal, (58), which is unfortunate given the physician's stress initiated by self-harm and suicidal behavior (59). This article thus provides some clues on how practitioners can address, as first-line professionals, such situations. Indeed, access to mental health professionals and facilities is not easy in many middle income European countries: this emphasizes the importance of the role of primary care practitioners in dealing with these situations, and deciding what immediate actions have to be taken to protect the adolescent. In addition, when a psychological or psychiatric investigation and treatment is set up, or when the adolescent is hospitalized, the primary care physician should offer follow-up meetings with the adolescent and the parents or caregivers to support the mental health care offered by specialists. Suicidal behavior are not rare in adolescents' life and always correspond to a suffering that needs to be addressed; however, fortunately, in almost all case, they do not end up with a death. Primary care providers and especially pediatricians can be instrumental in supporting their young patients and their parents to overcome such a crisis (54), especially given the high amount of trouble and suffering that the COVID pandemics has brought.

## Author Contributions

P-AM has written several drafts of the paper and worked on the references. AH and AM have proposed the topic of the paper and reviewed earlier versions. LM has worked on the content of the paper, including conceptual issues. A-EA and CK have worked on the different versions of the article. All authors contributed to the article and approved the submitted version.

## Conflict of Interest

The authors declare that the research was conducted in the absence of any commercial or financial relationships that could be construed as a potential conflict of interest.

## Publisher's Note

All claims expressed in this article are solely those of the authors and do not necessarily represent those of their affiliated organizations, or those of the publisher, the editors and the reviewers. Any product that may be evaluated in this article, or claim that may be made by its manufacturer, is not guaranteed or endorsed by the publisher.
